# Randomised phase II trial to investigate catumaxomab (anti-EpCAM × anti-CD3) for treatment of peritoneal carcinomatosis in patients with gastric cancer

**DOI:** 10.1038/s41416-018-0150-6

**Published:** 2018-07-10

**Authors:** Maren Knödler, Justus Körfer, Volker Kunzmann, Jörg Trojan, Severin Daum, Michael Schenk, Frank Kullmann, Sebastian Schroll, Dirk Behringer, Michael Stahl, Salah-Eddin Al-Batran, Ulrich Hacker, Stefan Ibach, Horst Lindhofer, Florian Lordick

**Affiliations:** 1 University Cancer Center Leipzig (UCCL), University Hospital Leipzig, Leipzig, Germany; 2 Department of Internal Medicine II, University Hospital Würzburg, Würzburg, Germany; 3 Department of Internal Medicine I, University Hospital Frankfurt, Frankfurt am Main, Germany; 4 Medical Department, Division of Gastroenterology, Infectiology and Rheumatology, University Hospital Berlin (Charite), Campus Benjamin Franklin, Berlin, Germany; 5 Department of Clinical Oncology and Hematology, Hospital Barmherzige Brüder Regensburg, Regensburg, Germany; 6 Department of Internal Medicine I, Hospital Weiden, Weiden, Germany; 7 Department of Internal Medicine III, Hospital Braunschweig, Braunschweig, Germany; 8 Department of Hematology, Oncology and Palliative Medicine, Augusta-Kranken-Anstalt, Bochum, Germany; 9 Department of Clinical Oncology and Hematology, Hospital Essen-Mitte Essen, Essen, Germany; 10 Department of Clinical Oncology and Hematology, Hospital Nordwest GmbH, Frankfurt, Germany; 11 WiSP Scientific Service Pharma GmbH, Langenfeld, Germany; 12 LINDIS Biotech GmbH, Planegg, Germany

**Keywords:** Gastric cancer, Cancer immunotherapy

## Abstract

**Background:**

Peritoneal carcinomatosis (PC) represents an unfavourable prognostic factor for patients with gastric cancer (GC). Intraperitoneal treatment with the bispecific and trifunctional antibody catumaxomab (EpCAM, CD3), in addition to systemic chemotherapy, could improve elimination of PC.

**Methods:**

This prospective, randomised, phase II study investigated the efficacy of catumaxomab followed by chemotherapy (arm A, 5-fluorouracil, leucovorin, oxaliplatin, docetaxel, FLOT) or FLOT alone (arm B) in patients with GC and PC. Primary endpoint was the rate of macroscopic complete remission (mCR) of PC at the time of second diagnostic laparoscopy/laparotomy prior to optional surgery.

**Results:**

Median follow-up was 52 months. Out of 35 patients screened, 15 were allocated to arm A and 16 to arm B. mCR rate was 27% in arm A and 19% in arm B (*p* = 0.69). Severe side effects associated with catumaxomab were nausea, infection, abdominal pain, and elevated liver enzymes. Median progression-free (6.7 vs. 5.4 months, *p* = 0.71) and overall survival (13.2 vs. 13.0 months, *p* = 0.97) were not significantly different in both treatment arms.

**Conclusions:**

Addition of catumaxomab to systemic chemotherapy was feasible and tolerable in advanced GC. Although the primary endpoint could not be demonstrated, results are promising for future investigations integrating intraperitoneal immunotherapy into a multimodal treatment strategy.

## Introduction

Peritoneal carcinomatosis (PC) is a common manifestation of relapse or primary metastatic spread in patients with gastric cancer (GC). Survival outcomes are poor.^1^ Due to variable drug delivery to the peritoneum, it is still unclear, to which extent systemic chemotherapy is effective for PC.^2^ Nevertheless, systemic chemotherapy is considered the standard treatment for PC.^3^ Effective strategies for treatment of PC are strongly needed. Besides to chemotherapy, local treatment approaches like cytoreductive surgery (CRS) plus hyperthermic intraperitoneal chemotherapy (HIPEC), which utilises surgery to reduce the visible tumour burden and HIPEC to eradicate peritoneal micrometastases are an option in treating PC in GC patients. Survival analyses after CRS plus HIPEC have shown that complete cytoreduction is associated with better overall survival (OS).^4^ However, a precise selection of patients (who should be in a good general condition, have a resectable primary gastric tumour and low peritoneal cancer burden) is highly recommended.^5^

Biologically targeted anti-cancer drugs in addition to systemic chemotherapy might be useful to treat PC. Catumaxomab (formerly marked by Fresenius Biotech, Munich, Germany) was developed and approved as a targeted therapy for the i.p. treatment of malignant ascites in cancers expressing the epithelial cell-adhesion molecule (EpCAM).^6^

EpCAM is physiologically expressed on epithelial tissues. In contrast, non-epithelial tissues like endothelium and mesenchymal tissues are EpCAM negative. Due to high-level expression on different epithelial tumours, EpCAM was considered as an interesting target for anticancer therapy.^7^ The cell surface protein is known to be overexpressed in >90% of gastric tumours.^7^ In case of peritoneal application for PC, catumaxomab exclusively binds to epithelial tumour cells and not to the EpCAM-negative mesothelial cells of the peritoneal surface. Catumaxomab is a chimeric (rat-murine) bispecific and trifunctional antibody (trAb), which combines the characteristics of classical monoclonal antibodies and bispecific molecules. TrAbs have two antigen binding sites with two different specificities. Catumaxomab in particular binds three different cell types: (1) one antigen binding site does recognise EpCAM,^8^ (2) the second antigen binding site connects to CD3 positive T cells^9^ and (3) the Fc region binds to type I, IIa, and III Fcγ receptors (FcγR) on accessory cells of the immune system initiating the activation of accessory cells (e.g., macrophages, dendritic cells, and natural killer cells).^10^ Catumaxomab’s antitumour effect is a result of a complex immune reaction at the tumour site involving T cell-mediated lysis, antibody-dependent cell-mediated cytotoxicity and phagocytosis.^8^ Clinical trials have shown efficacy and acceptable tolerability of catumaxomab as i.p. treatment of malignant ascites in patients with ovarian and non-ovarian epithelial cancers.^6,11^ In a pivotal phase II/III study, patients with EpCAM-positive cancer (ovarian and non-ovarian cancer) presenting with symptomatic malignant ascites requiring therapeutic paracentesis were randomised to receive paracentesis plus i.p. catumaxomab or paracentesis alone. Catumaxomab led to a significantly longer puncture-free-survival time (a composite endpoint of time free of paracentesis and OS time) and prolonged time to progression. Subgroup analyses indicated that patients with advanced GC may benefit from this therapy with regard to OS, while other pre-planned analyses (ovarian cancer patients and non-ovarian cancer patients) did not reveal subgroup-specific OS improvements.

Most commonly reported adverse side effects of i.p. catumaxomab treatment were infusion-related abdominal pain and signs of the proposed immunological mechanism of action (e.g., pyrexia, nausea and vomiting). These events were generally mild-to-moderate in intensity and mostly fully reversible.^6^

In summary, PC in GC is a significant health problem resulting in poor prognosis and with no specific standard treatment. New and more effective therapies for prevention and treatment of PC are urgently needed. This phase II study evaluated the role of i.p. administration of catumaxomab followed by standard systemic chemotherapy as new multimodal treatment approach for patients with GC and macroscopic PC. The main objective was to investigate the efficacy of catumaxomab by determination of the rate of macroscopic complete remissions (mCR) of PC after treatment with one cycle (four doses) of catumaxomab followed by six cycles of chemotherapy with 5-fluorouracil, leucovorin, oxaliplatin and docetaxel (FLOT).

## Patients and methods

### Study design and patients

This multicentre prospective, randomised, open-label phase II study was sponsored by the Arbeitsgemeinschaft Internistische Onkologie (AIO) Studien gGmbH, registered under accession number NCT01504256 at ClinicalTrials.gov and activated in 11 centres in Germany. The study aimed to investigate the efficacy and safety of systemic treatment with i.p. catumaxomab prior to FLOT chemotherapy or FLOT alone in patients with GC and PC at primary diagnosis. Patients aged >18 years with a histologically confirmed diagnosis of GC including oesophagogastric junction cancer (EGJ) type II and type III, according to Siewert’s classification were eligible if they had a macroscopic PC (stages P1-4 according to Gilly’s classification^12^), an Eastern Cooperative Oncology Group (ECOG) performance status of 0 or 1, medically fit for potential gastrectomy after primary systemic (and i.p.) treatment, adequate organ functions, and a life expectancy of at least 12 weeks. Selection criteria excluded patients with distant metastasis other than PC, clinically significant cardiovascular disease <1 year before enrolment, history of HIV infection or chronic hepatitis B or C, active and clinically serious infection and pre-existing neuropathy >grade 1.

### Study procedures and endpoints

Diagnostic laparoscopy was performed to define a baseline status of the extent of PC prior to enrolment. All lesions were photographically documented during laparoscopy. Documentation of the greatest diameter of the lesions in each of the four quadrants was required. Classification of PC was done according to the Gilly PC index^12^ and scored according Sugarbaker’s PC index (PCI).^13^ Patients were randomly assigned to one of two treatment arms in a 1:1 ratio. Patients allocated to arm A received i.p. catumaxomab administered over 3 h via a constant infusion pump system at escalating doses of 10, 20, 50, and 150 µg on days 0, 3, 7, and 10. Intravenous paracetamol 1000 mg was administered prior to catumaxomab for prophylaxis of cytokine-release associated symptoms. After 7 days the last infusion of catumaxomab, six cycles of FLOT chemotherapy (5-fluorouracil (2600 mg/m^2^ as 24 h infusion, d1), leucovorin (200 mg/m^2^, d1), oxaliplatin (85 mg/m^2^, d1), docetaxel (50 mg/m^2^, d1), q2wk) were administered. In arm B patients received six cycles of FLOT without prior catumaxomab. After completing catumaxomab plus chemotherapy or chemotherapy alone, imaging-based response assessment was done and evaluated locally according to RECIST version 1.1 criteria^14^ and a second diagnostic laparoscopy or laparotomy was recommended for evaluating disease response and assessing the possibility of resection. In case of complete resectability, resection of the primary tumour by gastrectomy and peritonectomy including all macroscopically involved parts of the peritoneum were recommended as a treatment option. Primary endpoint was the rate of macroscopic complete remission (mCR) of PC, defined as disappearance of all peritoneal lesions on CT and second laparoscopy (if done), based on the intention-to-treat approach. Secondary endpoints included surgical resection rate (R0, R1, R2), OS, disease-free survival, progression-free survival (PFS) as well as frequency and severity of adverse events. Follow-up every 12 weeks for up to 1 year was requested in order to assess survival-related secondary objectives. Adverse events were assessed according to the National Cancer Institute Common Terminology Criteria for Adverse Events (NCI-CTCAE) version 3.0.

### Statistical analysis

The objective of this randomised phase II trial was to find evidence that the catumaxomab arm has a superior response rate compared with chemotherapy alone. Based on the findings from trials with systemic chemotherapy only, the mCR rate after standard treatment was expected to be not higher than 5%. It was assumed, that this is similarly valid for PC. Thus, the experimental therapy arm including catumaxomab was to be rated as insufficiently active, if the true mCR rate was 5% or lower. The experimental therapy would be considered to be a highly promising candidate for further development, if the true mCR rate amounted to 20% or more. According to these parameters, and applying a standard two-stage phase II design by Simon,^15^ MiniMax option, *N* = 21 eligible patients had to be randomised into the experimental arm to achieve a power of 80% with a type I error of 0.1. This incorporated an interim analysis on the first 12 patients allowing stopping for futility. A similar number of patients were to be randomised to the reference arm, to control for selection bias. All parameters were evaluated in an explorative or descriptive manner. Any *p* values were considered to be descriptive, are two-sided and presented explicitly without referring to hypotheses or a significance level. mCR rate of PC was compared using Fisher’s exact test. PFS and OS were estimated by the Kaplan–Meier method and compared using the logrank test.

## Results

### Patient characteristics

**Fig. 1:**
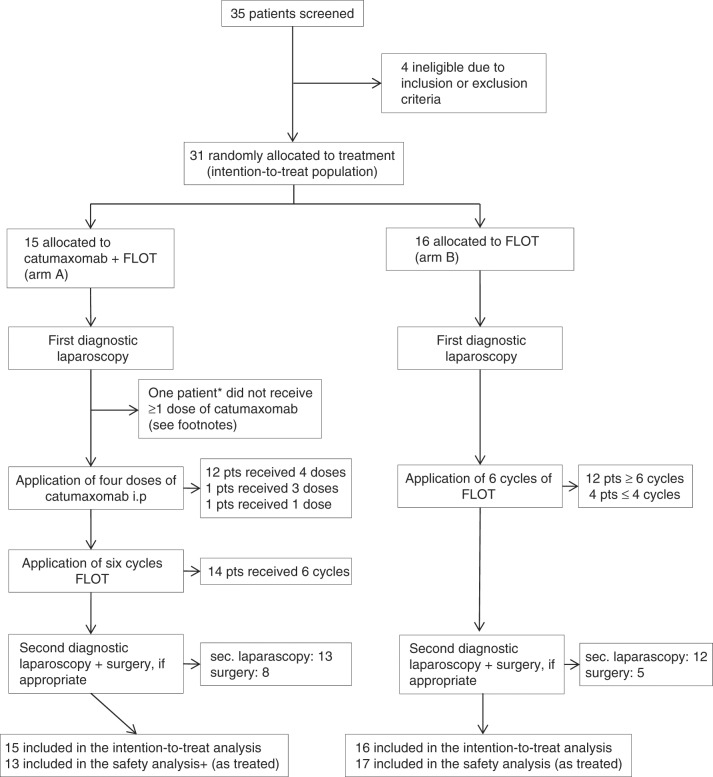
Trial profile depicting the total number of patients at screening, inclusion, randomisation, and analysis. FLOT 5-fluorouracil, leucovorin, oxaliplatin and docetaxel, i.p. intraperitoneal, pts patients. *one patinet did not receive at least one dose of catumaxomab and was switched to the FLOT arm (for safety population only). +one patient died after three doses of catumaxomab and did not receive FLOT treatment

Figure 1 shows the CONSORT diagram. Between October 2011, and December 2014, 35 patients were registered. When catumaxomab became no longer available in 2014 due to stop of production and marketing, the study protocol was amended accordingly and patient recruitment was stopped prematurely in December 2014. Four patients were excluded from analysis due to violation of selection criteria, as evaluated in a blinded pre-analysis, leaving 31 patients evaluable for final analysis. Of these, 15 were randomly allocated to catumaxomab plus FLOT (arm A) and 16 to FLOT only (arm B). Out of 15 patients allocated to arm A, 14 received at least one dose of catumaxomab. The full set of four applications of catumaxomab could be administered to 12 patients (86%). In arm A, 14 patients (100%) obtained six cycles of FLOT. In arm B, six cycles or more of systemic chemotherapy could be administered to 12 patients (75%). Table 1 delineates demographics and baseline disease characteristics.

**Table 1:** Baseline demographics and clinical characteristics of the study population

	Catumaxomab + FLOT (Arm A) *N* = 15	FLOT (Arm B) *N* = 16
Age, years		
Median (range, IQR)	56 (25–77, 49–63)	52 (38–73, 43–62)
Sex, male	7 (47%)	10 (62%)
ECOG performance status		
0	7 (50%)	8 (50%)
1	7 (50%)	7 (44%)
2	–	1 (6%)
Primary site		
Stomach	14 (93%)	11 (69%)
Oesophago-gastric junction	1 (7%)	5 (31%)
TNM-Classification		
T3	9 (60%)	10 (63%)
T4	4 (27%)	4 (25%)
Tx	2 (13%)	2 (12%)
N0	3 (20%)	3 (19%)
N+	10 (67%)	11 (69%)
Nx	2 (13%)	2 (12%)
M1 (Peritoneal carcinomatosis)	15 (100%)	16 (100%)
Gilly classification		
P1	1 (7%)	1 (6%)
P2 + P3	9 (60%)	10 (63%)
P4	5 (33%)	5 (31%)
Peritoneal Cancer Index (PCI)		
Median (range, IQR)	8 (1–34, 5–17)	12 (1–33, 3–19)

*IQR* interquartile range, *ECOG* Eastern Cooperative Oncology Group

### Efficacy

Four patients (27%) in the catumaxomab arm (exact 95% CI: 0.08–0.55) and three patients (19%) in the chemotherapy alone arm (exact 95% CI: 0.04–0.55) showed mCR after systemic treatment. This difference in favour of arm A was not statistically significant (*p* = 0.69). Second laparoscopy/laparotomy for response assessment was not carried out in three patients in arm A and five patients in arm B. Regarding the clinical response (according to RECIST criteria) to systemic treatment, a partial response rate of 46% was achieved in both arms (Table 2).

**Table 2:** Response rate

Macroscopic complete remission of peritoneal carcinomatosis	Catumaxomab + FLOT(*N* = 15)	FLOT(*N* = 16)
Complete remission (CR)	4 (27%)	3 (19%)
Non CR	9 (60%)	9 (56%)
No data	2 (13%)	4 (25%)
Clinical response (RECIST)	Catumaxomab + FLOT(*N* = 13)	FLOT(*N* = 13)
Partial response	6 (46%)	6 (46%)
Stable disease	3 (23%)	2 (15%)
Progressive disease	3 (23%)	5 (38%)
Not evaluated	1 (8%)	–

*CR* complete remission, *RECIST* response evaluation criteria in solid tumours

**Fig. 2:**
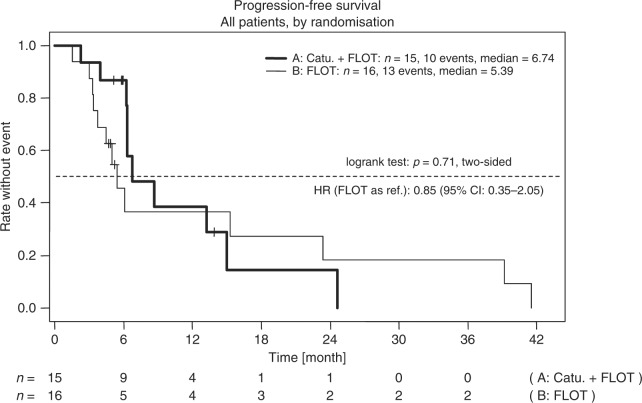
Kaplan–Meier estimates for progression-free survival in the intention-to treat population. FLOT 5-fluorouracil, leucovorin, oxaliplatin and docetaxel, Catu Catumaxomab; HR hazard ratio, CI confidence interval

**Fig. 3:**
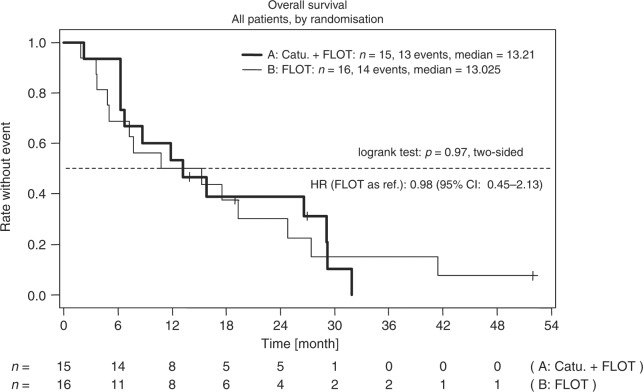
Kaplan–Meier estimates for overall survival in the intention-to treat population. FLOT 5-fluorouracil, leucovorin, oxaliplatin and docetaxel; Catu: Catumaxomab; HR: hazard ratio; CI: confidence interval

At time of analysis, median follow-up was 52 months. Ten patients in arm A (67%) and 13 patients in arm B (81%) had tumour progression. Median PFS was 6.7 months vs. 5.4 months in arm A vs. B (*p* = 0.71) (Fig. 2). Median OS was 13.2 months in arm A and 13.0 months in arm B (*p* = 0.97) (Fig. 3). At the time of evaluation, one patient in arm A (7%) and one patient in arm B (6%), respectively, were alive without tumour progression. Two patients (arm A: *N* = 2 (13%), arm B: *N* = 2 (12%)) in both treatment arms were still alive at the time of final analysis. Surgery of the primary tumour and peritonectomy were done in eight patients in arm A (53%), and five patients in arm B (31%). Reasons for no surgery were: early progression (28%), no possibility of R0 resection in the peritoneum (22%), or at other sites (22%) and impaired performance status (11%). Exploratory analysis showed that the PCI (dichotomised at the median) is a significant prognostic factor for PFS (*p* = 0.046). Patients who achieved a mCR in both arms were those with a PCI below the median.

### Safety

Severe side effects (grade III/IV adverse events) associated with catumaxomab treatment prior to chemotherapy (arm A) were nausea (15%), infection (23%), abdominal pain (31%), and elevated liver enzymes (gGT (31%), bilirubin (23%)). Four patients (29%), experienced SAE’s during catumaxomab treatment. Table 3 illustrates the reported adverse events in both arms that occurred during FLOT chemotherapy with a trend towards more adverse events following catumaxomab (arm A).

**Table 3:** Adverse events in the safety population during FLOT chemotherapy

	Catumaxomab + FLOT (Arm A)		FLOT (Arm B)	
	Any grade	Grade 3 or 4	Any grade	Grade 3 or 4
Adverse events				
Nausea	10 (77%)	1 (8%)	12 (71%)	3 (18%)
Vomiting	5 (38%)	1 (8%)	7 (41%)	2 (12%)
Diarrhoea	7 (44%)	0 (0%)	9 (53%)	1 (6%)
Fatigue	5 (38%)	1 (8%)	10 (59%)	1 (6%)
Hand-foot syndrome	4 (31%)	1 (8%)	4 (24%)	2 (12%)
Rash	3 (23%)	0 (0%)	3 (18%)	0 (0%)
Anaemia	13 (100%)	2 (16%)	13 (76%)	1 (6%)
Leukocytopaenia	11 (85%)	1 (8%)	14 (82%)	1 (6%)
Neutropaenia	12 (92%)	4 (33%)	14 (82%)	5 (32%)
Thrombocytopaenia	5 (38%)	0 (0%)	3 (18%)	0 (0%)
Pyrexia	4 (30%)	0 (0%)	4 (24%)	0 (0%)
Chills	7 (54%)	2 (15%)	11 (65%)	1 (6%)
Febrile neutropaenia	0 (0%)	0 (0%)	1 (6%)	1 (6%)
Infection	3 (23%)	2 (15%)	4 (24%)	1 (6%)
Allergic reaction	0 (0%)	0 (0%)	0 (0%)	0 (0%)
Mucositis/stomatitis	1 (8%)	0 (0%)	6 (35%)	1 (6%)
Pain	6 (46%)	0 (0%)	7 (41%)	1 (6%)
Dyspnoea	2 (15%)	0 (0%)	1 (6%)	0 (0%)
Alopecia	7 (54%)	0 (0%)	8 (47%)	0 (0%)
Elevated bilirubin	1 (8%)	0 (0%)	0 (0%)	0 (0%)
Serious adverse events				
Any	3 (23%)		5 (29%)	
None	10 (77%)		12 (71%)	

## Discussion

The aim of this study was to investigate the efficacy and safety of systemic treatment with i.p. catumaxomab in addition to systemic chemotherapy or chemotherapy alone in patients with GC and PC at primary diagnosis. Unfortunately, the primary endpoint of this study – improvement of the mCR rate of PC – was not met. A trend towards superiority of the experimental arm did not reach statistical significance (27% in arm A vs. 19% in arm B). However, the mCR rate in the standard arm B was significantly higher compared to the assumptions made for sample size calculation. Nonetheless, i.p. treatment with catumaxomab in this disease setting showed an acceptable safety profile and addition of catumaxomab to six cycles of FLOT chemotherapy did not lead to any unexpected adverse events: the toxicity profile was roughly the same as in previous studies where catumaxomab was mostly investigated in patients with malignant ascites.^16,17^ However, we observed a trend towards more adverse events during FLOT chemotherapy following catumaxomab (arm A) compared with chemotherapy alone (arm B), without preventing the feasibility of systemic chemotherapy. The most frequent severe side effects associated with catumaxomab were nausea, infection, abdominal pain, and elevated liver enzymes.

Median PFS was 6.7 months in arm A vs. 5.4 months in arm B and median OS was 13.2 months and 13.0. So we have to conclude that no major differences in survival outcomes between the two arms were seen. Of note, both the efficacy and safety assessment are limited by small patient numbers. However, the survival outcomes are within the expected range for a palliative treatment approach in patients with metastatic GC, though median OS in both arms was longer than expected in a stage IV GC population with primary PC.^18^ One may argue that this could be due to the selection of patients with PC only, without other distant disease manifestations, and therefore, probably a generally lower tumour burden.

Although the primary endpoint was not met, the results are important and support the notion that future prospective trials investigating the application of i.p. therapy as part of a multimodal treatment strategy are feasible. Another important teaching of the actual study could be that the sequence immunotherapy followed by chemotherapy within 1 week is counterproductive. This holds true especially using T cell redirecting bispecific antibodies, which are dependent on a functional immune system and where antitumour responses can last for weeks and even months.^19,20^ In the meantime, clinical investigations are available that the sequence, chemotherapy followed by immunotherapy, after a time period when the immune system has recovered from chemotherapy, is obviously better suited than vice versa.^21,22^ This fact should be taken into account for the design of upcoming studies using catumaxomab.

Other local treatment options in patients with PC are under investigation. Recent studies comparing CRS with CRS plus HIPEC have shown a small but statistically significant survival improvement.^23,24^ However, CRS and HIPEC have also relevant morbidity and side effects.^25^ Therefore, a careful selection of patients who might benefit from this approach is important and is still to be defined. Data regarding the effectiveness and benefit of CRS and HIPEC in patients with GC und PC are still limited. Further clinical research on this approach is needed.

Our study has several limitations. The major limitation is the relatively small number of included patients which is in part a consequence of the premature closure of the trial due to the study drug catumaxomab becoming unavailable during the conduct of the study. Linked to this unforeseen problem, we observed a non-ideal balance between the PCI scores in both study arms which might have been more balanced with a higher number of recruited patients. In addition, a priori stratification of patients according to PCI at the timepoint of randomisation might have been useful. Another limitation is the lack of biological correlative analyses. It would be important to understand which subpopulation of patients benefits from i.p. catumaxumab. Nevertheless, we could demonstrate that a local treatment approach in patients with peritoneal metastatic GC seems to be feasible and should undergo further evaluation in clinical trials. Due to the low number of patients having isolated peritoneal metastases, conduction of clinical trials evaluating local treatment approaches in GC is challenging. However, we believe that for patients in a good performance status and with controlled disease during a defined time interval of systemic chemotherapy (e.g., 3–4 months) local therapy of peritoneal metastases deserves further evaluation.

In conclusion, the achievement of the primary endpoint of the study (improvement of the macroscopic CR rate of PC) unfortunately could not be demonstrated. In addition, no major differences in survival outcomes between the two arms were seen. However, i.p. immunotherapy in this disease setting showed an acceptable safety profile and addition of i.p. catumaxomab to systemic chemotherapy did not lead to any unexpected adverse events. The main achievement of this study, therefore was to demonstrate that the addition of an i.p. immunotherapy as part of a multimodal treatment approach was feasible in a multicentre setting and tolerable for patients with PC from GC.

## Availability of data and material

The sponsor (AIO) was responsible for data management and statistical analysis and was supported by a contracted clinical research organisation (WiSP GmbH, Langenfeld). The first authors and the senior author had full access to all study data and MK and FL had the final responsibility for the decision to submit for publication.
